# Racial and Ethnic Disparities in the Outcomes and Treatment of Patients Admitted with Heart Failure: A Nationwide Analysis

**DOI:** 10.3390/jcm14010018

**Published:** 2024-12-24

**Authors:** Nahush Bansal, Abdulmajeed Alharbi, Shuhao Qiu, Libin Wang

**Affiliations:** 1 Department of Internal Medicine, The University of Toledo, Toledo, OH 43606, USA; 2 Division of Cardiovascular Medicine, Tufts Medical Center, Boston, MA 02111, USA

**Keywords:** heart failure, racial disparities, treatment gap, mortality, cardiac resynchronization therapy, ventricular assist device, heart transplant, racial inequality

## Abstract

**Background/Objectives**: Heart failure is the leading cause of hospital admission and mortality. Racial disparities have been demonstrated in various cardiovascular disorders; however, the data for in-hospital outcomes, complications, and procedural rates are limited. **Methods**: Utilizing the National Inpatient Sample (NIS) database, this retrospective cohort study included adult patients admitted with a principal diagnosis of heart failure. Coding for race and ethnicity in the NIS combines self-reported race and ethnicity provided by the data source into 1 data element (“RACE”). We compared the outcomes between various racial groups, focusing on mortality, the length of stay (LOS), hospital charges, and complications. Differences in the utilization of advanced therapies, including implantable cardiac defibrillators, cardiac resynchronization therapy (CRT), ventricular assist devices (VADs), and heart transplant, were also analyzed. **Results**: Out of 1,107,860 patients hospitalized with heart failure, 715,345 (64.57%) patients were White, 244,394 (22.06%) patients were Black, and 97,063 (8.31%) patients were Hispanic. Compared to White people, the odds of in-hospital mortality were lower among Black (aOR 0.74; 95% CI 0.68–0.81; *p* < 0.001) and Hispanic (aOR 0.78; 95% CI 0.69–0.88; *p* < 0.001) people. Complication rates including cardiogenic shock were found to be significantly lower in Black people (aOR 0.86; 95% CI 0.77–0.96; *p* < 0.001) and in Hispanic (aOR 0.72; 95% CI 0.63–0.81; *p* < 0.001) people. The rates of acute respiratory failure were also lower in Black (aOR 0.72; 95% CI 0.69–0.74; *p* < 0.001) and Hispanic (aOR 0.77; 95% CI 0.73–0.81; *p* < 0.001) people as opposed to White people. However, Black people were found to have higher rates of acute kidney injury (aOR 1.11; 95% CI 1.07–1.14; *p* < 0.001) and cardiac arrest (aOR 1.17; 95% CI 1.03–1.34; *p* = 0.02) compared to White people. Black people were less likely to receive advanced interventions, including cardiac resynchronization therapy (aOR 0.71; 95% CI 0.60–0.83; *p* < 0001), a ventricular assist device (aOR 0.45; 95% CI 0.34–0.59; *p* < 0.001), and heart transplants (aOR 0.57; 95% CI 0.42–0.77; *p* < 0.001), than White people. Hispanic people were found to have lower rates of ventricular assist device (aOR 0.49; 95% CI 0.33–0.72; *p* < 0.001) use than White people. **Conclusions**: These findings highlight significant racial disparities in mortality, secondary outcomes, and advanced therapy utilization in heart failure admissions. Further research is needed to identify the root factors for these disparities in order to guide targeted interventions to reduce this racial gap.

## 1. Introduction

With an estimated prevalence of 6.7 million adults in the United States (U.S.) above the age of 20 years, heart failure is one of the leading causes of hospitalization and mortality [1]. The projected lifetime risk of heart failure is at 24%, with approximately 8.5 million cases forecast by 2030 in the U.S. Racial and ethnic disparities have been demonstrated to be a significant issue in the healthcare affecting patient outcomes, including those relating to cardiovascular disorders [2,3].

Healthcare disparities have been defined as racial and ethnic differences in the quality of healthcare and are attributable to the operation of healthcare systems and recognized or unrecognized bias or discrimination [4]. This includes differences in disease burden, the allocation of resources, and outcomes. Racial and ethnic minorities have been shown to be disproportionately impacted in heart failure care, management, and outcomes in various settings and regions [5,6]. However, large-scale data analyzing racial inequities for the in-hospital outcomes in heart failure admissions are limited.

Using the National Inpatient Sample (NIS) database, which represents all nonfederal acute care hospitals in the U.S., this study evaluates the racial and ethnic differences in outcomes, including mortality, length of stay (LOS), hospitalization charges, and complications in patients with heart failure. The utilization and occurrence of advanced heart failure therapies, including heart transplants, were also compared.

## 2. Materials and Methods

This study is a retrospective cohort analysis of adult patients admitted to acute care hospitals in the United States during 2020. Data were obtained from the National Inpatient Sample (NIS), a database developed by the Agency for Healthcare Research and Quality (AHRQ). The NIS is the largest publicly accessible all-payer inpatient database, covering all non-federal acute care hospitals across the country. The dataset employs stratified sampling based on factors such as hospital ownership, control, size, teaching status, geographic location, and urban or rural designation. A 20% probability sample of hospitals is drawn from each stratum, and the collected discharge data are appropriately weighted to generate national estimates. The 2020 dataset incorporates information from 49 statewide organizations, representing 98% of the U.S. population and offering comprehensive details at both the hospital and patient levels.

Patients chosen for inclusion in this study were identified using the International Classification of Diseases, Tenth Revision, Clinical Modification (ICD-10-CM) coding system. Codes for the principal diagnosis of heart failure (I50.xx, I09.81, I11.0, I13.0, I13.2) were selected to include all adult patients admitted primarily for heart failure. These specific codes demonstrated high validity and positive predictive value in prior research [7,8,9,10,11]. The primary risk factors analyzed were race and ethnicity, which were grouped into three categories: Black, Hispanic, and White. The NIS combines self-reported race and ethnicity into a single variable (“RACE”). In cases where both race and ethnicity were available, ethnicity took precedence when setting the HCUP value for RACE [12]. Patients belonging to other racial or ethnic groups (e.g., American Indian, Asian, or Pacific Islander patients) were excluded from the analysis due to small sample sizes, which limited our ability to draw meaningful conclusions for these populations. The primary outcome was in-hospital mortality, while secondary outcomes included length of stay (LOS), total hospital charges, and the occurrence of complications such as cardiac arrest, cardiogenic shock, acute kidney injury, and acute respiratory failure. Additionally, we evaluated the rates of advanced heart failure procedures, including implantable cardiac defibrillator (ICD) use, cardiac resynchronization therapy (CRT), ventricular assist device (VAD) use, and heart transplants, using the corresponding ICD-10 codes [6]. A comprehensive list of diagnostic and procedural codes is available in Supplementary Table S1.

Data analysis was performed using STATA/MP version 18.0 (Stata Corp, College Station, TX, USA). Baseline characteristics, such as comorbidities, primary payer, household income, hospital location, geographic region, and teaching status, were compared among Black, Hispanic, and White patients with heart failure. To identify significant associations, a univariate analysis was initially conducted for different outcomes. For adjusted comparisons, a multivariate logistic regression model was used to account for confounders, including age, sex, race, median household income, Charlson comorbidity index (CCI), hospital location (urban or rural), geographic region (Northeast, Midwest, West, or South), hospital teaching status, hospital bed size, and insurance status. Continuous variables were presented as means with 95% confidence intervals (CIs), and regression analysis was applied to assess differences between racial groups. For categorical variables, comparisons were performed using the chi-squared test. A two-sided *p* value < 0.05 was considered statistically significant for all analyses.

## 3. Results

### 3.1. Patient Characteristics

The NIS 2020 database included a total of 32,355,827 hospitalizations, of which 1,107,860 were for patients with a primary diagnosis of heart failure. Among these, 64.57% (715,345) were White patients, 22.06% (244,394) were Black patients, and 8.31% (92,063) were Hispanic patients. Smaller racial and ethnic groups included American Indian people (2.13%), Asian people or Pacific Islanders (0.58%), and other races (2.35%). The distribution and disparities in the minor racial groups and ethnicities are represented in the Supplementary Section (Table S2; Figure S1).

**Figure 1:**
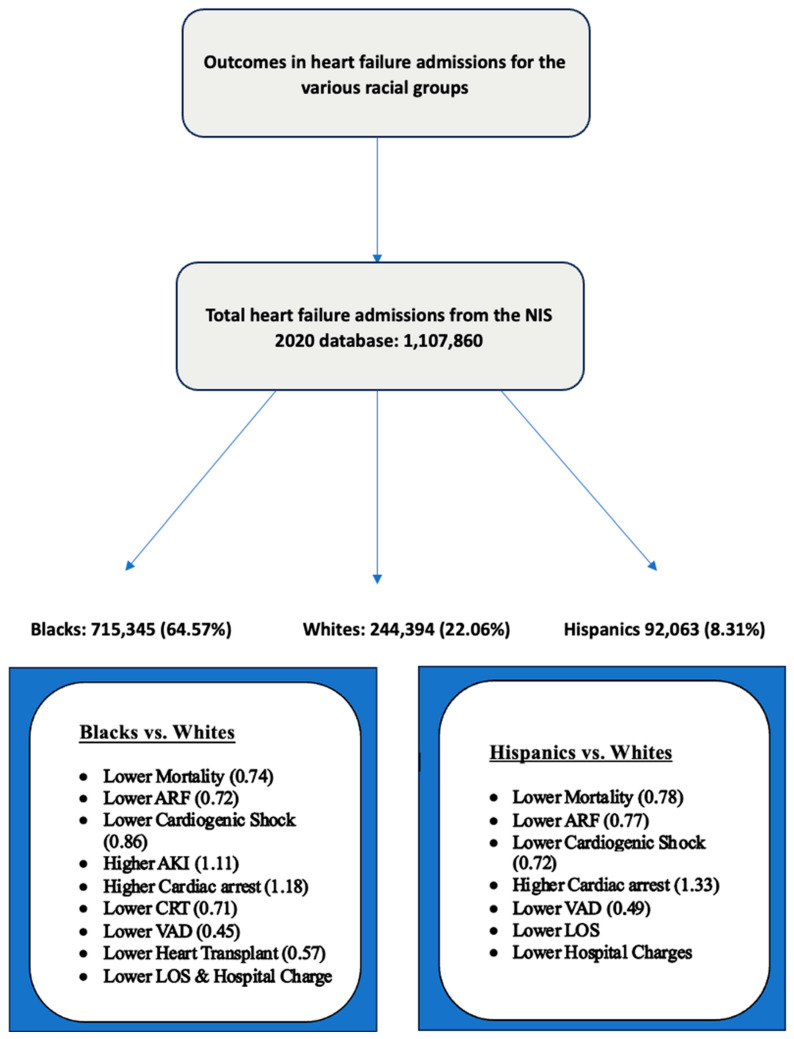
Significant racial disparities in the major outcomes for patients admitted with heart failure (ARF: acute respiratory failure; AKI: acute kidney; CRT: cardiac resynchronization therapy; VAD: ventricular assist device; LOS: length of stay). Adjusted odds ratios are presented in parentheses.

White patients were significantly older (mean age: 73.56 years, *p* < 0.001) compared to Black patients (mean age: 63.25 years) and Hispanic patients (mean age: 67.06 years). Key differences were also noted in comorbidities, primary payer, income levels, hospital teaching status, location, and region (Table 1). A higher proportion of Black (55.49%) and Hispanic patients (40.97%) belonged to the lowest quartile of median household income, and uninsurance rates were also higher among Black (5.03%) and Hispanic (5.68%) patients compared to Whites (2.26%). Regarding comorbidities, Black and Hispanic patients had higher rates of chronic kidney disease (CKD) and diabetes (both uncomplicated and complicated), whereas White patients exhibited a higher prevalence of acute myocardial infarction. The observed outcomes are visually summarized in Figure 1.

### 3.2. Primary Outcome: Mortality

The total in-hospital mortality for patients admitted with heart failure was 2.74% (30,385 patients). White people had the highest in-hospital mortality (3.09%), followed by Hispanic patients (2.23%) and then Black patients (1.83%). In adjusted models, the risk of in-hospital mortality was significantly higher among White patients as opposed to Black (aOR 0.74; 95% CI 0.68–0.81; *p* < 0.001) and Hispanic (aOR 0.78; 95% CI 0.69–0.88; *p* < 0.001) people. Table 2 shows the unadjusted outcomes for various racial groups regarding patients admitted with heart failure. Table S3 also shows propensity matched analysis results, consistent with the logistic regression. 

In addition to the multivariate logistic regression used in the primary analysis for mortality, we conducted a Cox proportional hazards regression to account for time-to-event outcomes, using length of stay (LOS) as the time variable. The findings of the Cox model were consistent with those of the logistic regression, reaffirming the observed racial and ethnic disparities in outcomes. However, given the limitations of using LOS as the sole time variable in the NIS database, we opted to focus on logistic regression in the main analysis, as it aligns better with the study design. The results of the Cox proportional hazards model are provided in the Supplementary Section (Table S4).

### 3.3. Secondary Outcomes

#### 3.3.1. Resource Utilization and Complications

Resource utilization was analyzed by determining the length of stay and hospital charges. In the adjusted models, the mean LOS was found to be slightly lower for Black patients (mean decrease 0.22 days; *p* < 0.001) and Hispanic patients (mean decrease 0.36 days; *p* < 0.001). Similarly, Black (mean decrease $3242; *p* < 0.001) and Hispanic (mean decrease $7624; *p* < 0.001) patients were found to have lower hospitalization charges than White patients (Table 3).

After adjusting for patient- and hospital-level confounders, Black and Hispanic patients had lower rates of cardiogenic shock (aOR 0.85, 95% CI 0.77–0.96, *p* = 0.007 and aOR 0.72, 95% CI 0.63–0.81, *p* < 0.001, respectively) and acute respiratory failure (aOR 0.72, 95% CI 0.69–0.74, *p* < 0.001 and aOR 0.77, 95% CI 0.73–0.81, *p* < 0.001, respectively). Black (aOR 1.18; 95% CI 1.03–1.34; *p* = 0.015) and Hispanic (aOR 1.33; 95% CI 1.12–1.59; *p* < 0.001) patients also had significantly higher rates of cardiac arrest than White people. Black people (aOR 1.11; 95% CI 1.07–1.14; *p* < 0.001) were also found to have higher acute kidney injury than White patients. 

#### 3.3.2. Interventions and Procedures

**Figure 2:**
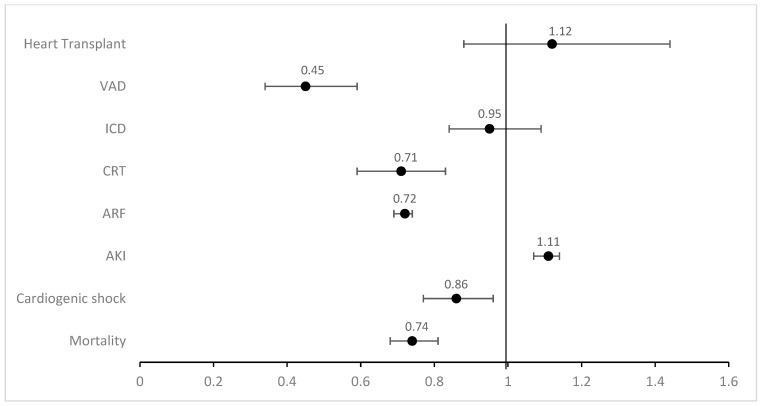
Adjusted odds ratio (aOR) for various categorical outcomes regarding Black vs. White ethnicity for patients admitted with heart failure (VAD: ventricular assist device; ICD: implantable cardiac defibrillator; CRT: cardiac resynchronization therapy; ARF: acute respiratory failure; AKI: acute kidney injury).

**Figure 3:**
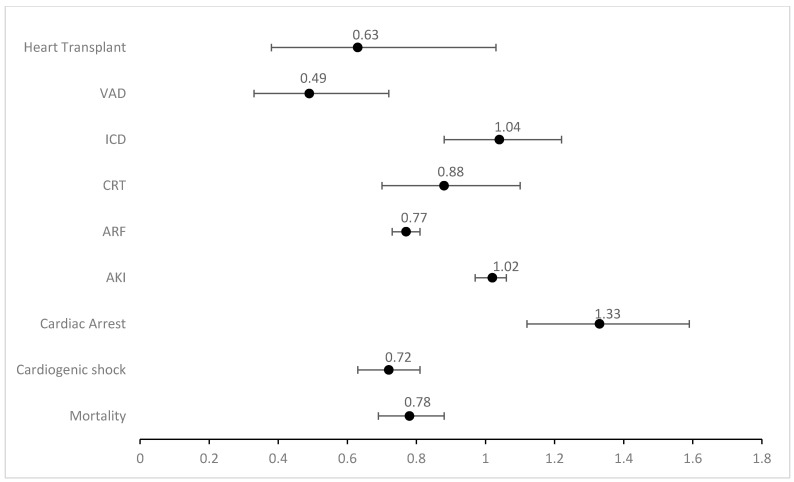
Adjusted odds ratio (aOR) for various categorical outcomes in Hispanic vs. White ethnicity for patients admitted with heart failure (VAD: ventricular assist device; ICD: implantable cardiac defibrillator; CRT: cardiac resynchronization therapy; ARF: acute respiratory failure; AKI: acute kidney injury).

Black (aOR 0.45; 95% CI 0.34–0.59; *p* < 0.001) and Hispanic (aOR 0.49; 95% CI 0.33–0.72; *p* < 0.001) patients were found to have lower rates for the use of ventricular assist devices compared to White patients. Black patients also had significantly lower rates of cardiac resynchronization therapy (aOR 0.70; 95% CI 0.59–0.83; *p* < 0.001) and heart transplants (aOR 0.57; 95% CI 0.42–0.77; *p* < 0.001) as opposed to White patients. No significant differences were noticed in various racial groups for implantable cardiac defibrillators. Figure 2 shows the adjusted outcomes between Black and White patients and Figure 3 shows the adjusted outcomes between Hispanic and White patients. Table 4 illustrates these results with confidence intervals and *p* values.

#### 3.3.3. Predictors of In-Hospital Mortality

In a multivariate analysis, factors independently associated with increased in-hospital mortality in patients admitted with heart failure were older age, a higher Charlson comorbidity index score, and a larger hospital bed size. Table 5 illustrates these findings.

## 4. Discussion

In this large nationwide study analyzing the racial disparities among heart failure admissions, Black and Hispanic patients were found to have lower rates of mortality as compared to White patients. White patients also had higher rates of complication, including cardiogenic shock and acute respiratory failure. On the contrary, acute kidney injury and cardiac arrest were found to be higher in the Black and Hispanic racial groups. Another key finding was the existence of racial disparities in receiving advanced therapies like CRT, ventricular assist device, and heart transplant, with lower rates seen in Black and Hispanic people.

This study showed higher in-hospital mortality for White patients as opposed to Black and Hispanic patients. These results were consistent with previous studies. In a large study of Medicare beneficiaries above the age of 65 years admitted with heart failure, Black and Hispanic patients were less likely to die in the hospital as opposed to White patients [13]. Regional studies have also shown lower one-year mortality post-hospitalization in Black and Hispanic patients [5,6]. In this study, White patients were also found to have significantly higher rates for serious complications, including acute respiratory failure and cardiogenic shock.

Various pathophysiological, socioeconomic, and healthcare access factors can potentially explain racial disparities in mortality and morbidity. The underlying etiology and pathophysiology for heart failure differs between various racial groups. Black populations are more likely to develop heart failure secondary to comorbid conditions like hypertension and diabetes, whereas White patients are more vulnerable to heart failure from coronary artery disease and ischemic cardiomyopathy [14,15]. Studies have shown that ischemic etiologies for heart failure have higher odds of cardiovascular death as opposed to nonischemic etiologies [16]. Black and Hispanic patients were found to be significantly younger than White patients in our cohort, potentially explaining the favorable outcomes noticed in these racial groups. Previous research has also shown that Black and Hispanic patients tend to be healthier than Whites [5,17]. The potential contribution of baseline demographic and health status to explaining the racial disparities noticed in heart failure admissions likely just plays a minor role at best, especially since the outcomes in our study were adjusted for these factors. However, the accurate determination of the severity of primary and comorbid diseases is limited in NIS, as NYHA class, lab and clinical parameters, and echocardiographic parameters cannot be determined using the database.

Black and Hispanic patients were found to undergo lower rates of advanced heart failure therapies, including cardiac resynchronization therapy, the use of ventricular assist devices, and heart transplants, than White patients. These disparities in the utilization of the advanced therapies are likely contributed to by the differences in socioeconomic status. Our cohort showed that Black and Hispanic patients were more likely to be uninsured and have less Medicaid coverage than White patients. This was despite the fact that and Hispanic people had significantly higher numbers of people in the lower income bracket than White people. In this study, White people were found to have high mortality rates despite the greater use of advanced heart failure therapies. This paradox may be explained by the severe and refractory nature of the disease in White people. Nonetheless, these findings are concerning as they show the existence of racial disparity in accessibility to advanced therapies. A study by Chouairi et al. demonstrated that Black and Hispanic people are less likely to undergo heart transplant procedures, despite the increasing proportions of these racial groups on the list of transplant patients [18]. Black and Hispanic people were also shown to be less likely to receive heart transplant when they were patients with cardiogenic shock secondary to peripartum cardiomyopathy [19].

Higher rates of complications and the higher use rate of advanced therapies explain greater resource utilization noticed in our study for White people, in terms of length of stay and hospitalization charges, as compared to Black and Hispanic people. The poor kidney reserve seen in Black patients due to the underlying high rates of chronic kidney disease, as seen in the demographic profile of our cohort, potentially explains the high rates of acute kidney injury in this population. Consistent with multiple previous studies, Black people were found to have higher rates of cardiac arrest than White people [20,21]. However, no racial disparities were seen in implantable cardiac defibrillator placement in our study, which was contrary to previous research [22]. This finding likely indicates the narrowing of racial gaps in receiving ICD counseling and placement. However, more large-scale and directed studies are needed to verify this claim.

Findings from the study indicate the urgent need for more research to understand the factors responsible for the racial and ethnic disparities observed in patients admitted with heart failure. Specifically, it is important to determine the proportion of disparities caused by racial inequities in comparison to biological factors. Understanding the biases in the allocation of advanced heart failure therapies can help in performing targeted interventions to close the gap in terms of differential access to treatment modalities. Multidisciplinary and culturally tailored interventions, both at the level of organization and individual, are likely to play an essential role in reducing these racial disparities.

It is important to note that the National Inpatient Sample (NIS) lacks granular clinical details such as QRS duration, left ventricular ejection fraction (LVEF), or NYHA classification, which are critical for determining eligibility for advanced therapies like ICD, CRT, or VAD. Therefore, the lower rates of these procedures observed in certain racial groups may not necessarily indicate undertreatment, but could reflect differences in clinical indications. This limitation highlights a significant gap in evidence and underscores the need for future research, using datasets with detailed clinical parameters, to better delineate whether disparities arise from differences in eligibility, healthcare access, or provider bias.

Our study has several limitations. First, as a retrospective analysis that relies on ICD-10 codes, there is a potential for misclassification bias. However, prior studies have demonstrated that ICD-10 coding for heart failure has high accuracy and positive predictive value [10]. Additionally, the use of an administrative database like the NIS limits our ability to assess the severity of illness accurately. To address this, we incorporated the Charlson Comorbidity Index (CCI), a well-validated prognostic tool [23]. It is also important to note that certain clinical parameters, such as laboratory values, echocardiographic findings, heart failure etiology, and specific causes of mortality, cannot be captured through the NIS. Furthermore, the database does not include information on readmissions related to heart failure, which could provide additional insights. Despite these limitations, the NIS provides a large, nationally representative sample with robust administrative data, offering sufficient statistical power to draw meaningful conclusions about disparities in outcomes across racial and ethnic groups.

## 5. Conclusions

This study showed racial disparities in heart failure patients. White patients were found to have higher rates of mortality, resource utilization and serious complications, including acute respiratory failure and cardiogenic shock, than Black and Hispanic patients. Minority racial groups were found to be less likely to receive advanced heart failure therapies including cardiac resynchronization therapy, ventricular assist devices, and heart transplants. These findings indicate the existence of racial inequity in the outcomes and in the management of patients admitted with heart failure. Further research is needed to understand the biases and factors contributing to this racial disparity in order to make targeted interventions.

## Figures and Tables

**Table 1:** Demographics, hospital characteristics, and comorbidities in heart failure admissions stratified by racial groups.

	Whites	Blacks	Hispanics	*p* Value *
Total (%) of heart failure admissions	63	23.2	8.8	
Age (median)	76	63	68	<0.01
Female gender (%)	46.56	47.36	44.74	<0.01
Median Income in patients Zip code (%)				<0.01
$1–47,999	26.94	55.49	40.97	
$48,000–60,999	30.08	22.43	26.5	
$61,000–81,999	24.02	13.59	20.85	
≥$82,000	18.96	8.49	11.68	
Charlson comorbidity index (%)				0.12
1	8.98	7.48	7.5	
2	15.07	12.55	13.72	
3 or more	75.95	79.97	78.78	
Hospital Region				<0.01
Northeast	19.05	14.47	17.94	
Midwest	26.02	20.31	6.4	
South	39.02	56.14	37.67	
West	15.91	9.08	37.99	
Hospital Bed size (%)				<0.01
Small	25.26	21.87	20.72	
Medium	29.06	27.09	30.8	
Large	45.68	51.04	48.48	
Hospital Location (%)				<0.01
Rural	12.96	6.12	2.38	
Urban	87.04	93.88	97.62	
Hospital Teaching (%)				<0.01
Non-teaching (%)	33.63	20	22.45	
Teaching (%)	66.37	80	77.55	
Insurance type (%)				<0.01
Medicaid	78.98	58.49	60.39	
Medicare	7.27	21.79	23.45	
Private	11.48	14.7	10.48	
Uninsured	2.26	5.03	5.68	
Co-morbidities (%)				
Diabetes	12.76	13.46	16.62	<0.01
Diabetes with complications	34.48	39.85	46.95	<0.01
Renal dysfunction	52.32	61.01	58.41	<0.01
Myocardial infarction	23.1	19.58	21.69	<0.01
Coronary artery disease (CAD)	49.28	37.34	43.88	<0.01
Atrial fibrillation	53	29.21	34.13	<0.01
Mitral regurgitation	6.91	6.68	6.74	0.31
Aortic stenosis	6.75	2.23	4.46	0.06

* *p* value ≤ 0.05 indicates significance.

**Table 2:** Unadjusted primary and secondary outcomes stratified by race and ethnicity in heart failure admissions.

Variable	Whites	Blacks	Hispanics	*p* Value
Deaths (%)	3.09	1.83	2.23	<0.01
Complications (%)				
Cardiogenic shock	2.91	3.55	2.82	<0.01
Cardiac arrest	0.73	0.89	1	<0.01
Acute kidney injury	35.31	38.65	37.75	<0.01
Acute respiratory failure	39.06	30.5	33.9	<0.01
Resource Use				
LOS, d	5.52 (5.46–5.59)	5.74 (5.61–5.86)	5.43 (5.28–5.59)	0.34
Hospital cost, $	58,829 (56,979–60,679)	64,181 (61,218–67,143)	78,837 (74,543–83,132)	0.27
Advanced Procedures (%)				
Implantable defibrillator	0.85	1.09	1.06	0.12
CRT	0.63	0.5	0.57	0.06
Ventricular assist device	0.23	0.16	0.16	<0.01
Heart transplant	0.15	0.16	0.16	0.68

Data presented as mean (range) and percentages. LOS: length of stay; CRT: cardiac resynchronization therapy.

**Table 3:** Racial disparities in adjusted quantitative outcomes in heart failure.

Quantitative Outcomes	Whites	Blacks	Hispanics	*p* Value
Length of stay (days)	5.52	5.30	5.16	<0.01
Hospitalization charges ($)	58,829	55,587	50,965	<0.01

**Table 4:** Adjusted with confidence intervals among various racial groups in patients with heart failure.

Outcomes (Blacks vs. Whites)	aOR; 95% CI	*p* Value
Mortality	0.74 (0.68–0.81)	<0.001 *
Cardiogenic shock	0.86 (0.77–0.96)	0.007 *
Cardiac arrest	1.18 (1.03–1.34)	0.02 *
Acute kidney injury	1.11 (1.07–1.14)	<0.001 *
Acute respiratory failure	0.72 (0.69–0.74)	<0.001 *
CRT	0.71 (0.59–0.83)	<0.001 *
ICD	0.95 (0.84–1.08)	0.47
VAD	0.45 (0.34–0.59)	<0.001 *
Heart transplant	0.57 (0.42–0.77)	<0.001 *
Outcomes (Hispanics vs. Whites)		
Mortality	0.78 (0.69–0.88)	<0.001 *
Cardiogenic shock	0.72 (0.63–0.81)	<0.001 *
Cardiac arrest	1.33 (1.12–1.59)	0.001 *
Acute kidney injury	1.01 (0.96–1.06)	0.63
Acute respiratory failure	0.77 (0.73–0.81)	<0.001 *
CRT	0.88 (0.70–1.1)	0.26
ICD	1.04 (0.88–1.22)	0.63
VAD	0.49 (0.33–0.72)	<0.001 *
Heart transplant	0.63 (0.38–1.02)	0.12

VAD: ventricular assist device; ICD: implantable cardiac defibrillator; CRT: cardiac resynchronization therapy. * *p* value lower than 0.05 demonstrates significance.

**Table 5:** Multivariate regression analysis with adjusted OR for in-hospital mortality.

Variable	Adjusted Odds Ratio (aOR)	*p* Value
Age	1.03 (1.02–1.05)	<0.01
Female sex	0.88 (0.83–0.93)	<0.01
Charlson comorbidity index	1.10 (1.08–1.12)	<0.01
Income quartile		
$1–47,999	Reference	
$48,000–60,999	1.03 (0.96–1.11)	0.36
$61,000–81,999	0.96 (0.88–1.05)	0.35
≥$82,000	0.97 (0.88–1.07)	0.6
Hospital location		
Rural	Reference	
Urban	0.98 (0.87–1.11)	0.85
Hospital bed size		
Small	Reference	
Medium	1.06 (0.97–1.16)	0.223
Large	1.29 (1.19–1.40)	<0.01

All ORs are adjusted for the other covariates listed. ORs > 1 indicate greater odds of all-cause in-hospital mortality.

## Data Availability

Data Availability Statements are available upon request, through the corresponding author.

## Supplementary Materials

The following supporting information can be downloaded at: https://www.mdpi.com/article/10.3390/jcm14010018/s1, Figure S1: Chart representing the distribution among the excluded/minority racial groups.; Table S1: ICD 10-CM codes used for various conditions and procedures in the study; Table S2: Unadjusted Primary and secondary outcomes stratified by minor racial groups in heart failure admissions; Table S3: Propensity matched analysis results for racial disparities for major outcomes in heart failure patients; Table S4: Cox proportional Hazards regression results for hospital mortality by racial groups.
