# Supplementary material for: Racial and Ethnic Disparities in the Outcomes and Treatment of Patients Admitted with Heart Failure: A Nationwide Analysis
Source: J Clin Med. 2024 Dec 24;14(1):18. doi: 10.3390/jcm14010018 (PMC11722288; doi:10.3390/jcm14010018)
Supplement: Supplementary file 1 [file jcm-14-00018-s001.zip › jcm-3338432-supplementary.pdf]

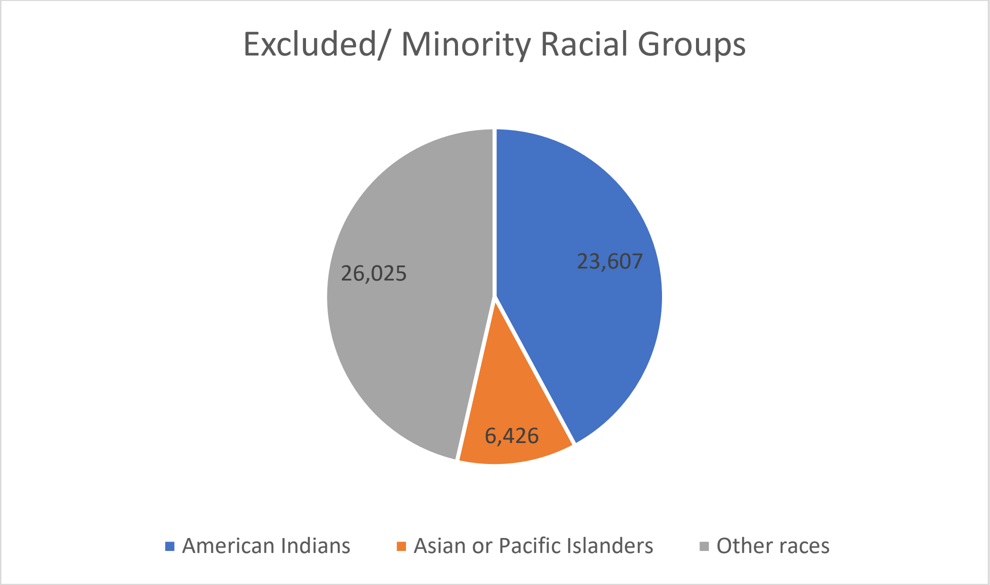

Figure S1. Chart representing the distribution among the excluded/minority racial groups.

Table S1. ICD 10-CM codes used for various conditions and procedures in the study.

**Condition**	**ICD10-CM codes**
Heart Failure	I50xx, I0981, I110, I130, I132
Cardiac arrest	I462, I468, I469
Cardiogenic shock	R570
Acute Kidney Injury	N170, N171, N172, N178, N179
Acute Respiratory Failure	J9600, J9601, J9602, J9620, J9621, J9622, J9690, J9691, J9692
Cardiac resynchronization therapy	0JH607Z, 0JH609Z, 0JH637Z, 0JH639Z,0JH807Z, 0JH809Z, 0JH837Z, 0JH839Z
Implantable cardiac defibrillator	02H40KZ, 02H43KZ, 02H44KZ, 02H60KZ, 02H63KZ, 02H64KZ, 02H70KZ, 02H73KZ, 02H74KZ, 02HK0KZ, 02HK3KZ, 02HK4KZ, 02HL0KZ, 02HL3KZ, 02HL4KZ, 02HN0KZ, 02HN3KZ, 02HN4KZ, 0JH608Z, 0JH60FZ, 0JH638Z, 0JH639Z, 0JH63FZ, 0JH808Z, 0JH838Z
Ventricular Assist Device	5A0211D, 5A0221D, 5A1522H, 02HA3RZ, 02HA3RJ, 02HA4RJ"
Heart Transplant	02YA0Z0, 02YA0Z1, 02YA0Z2

Table S2. Unadjusted Primary and secondary outcomes stratified by minor racial groups in heart failure admissions.

**Variable**	**American Indians**	**Asian or Pacific Islanders**	**Other Races**	***P* value**
**Deaths (%)**	2.69	3.79	2.83	0.13
**Complications (%)**				
Cardiogenic shock	4.16	4.26	3.83	0.66
Cardiac arrest	0.91	0.71	1.12	0.35
Acute Kidney Injury	41.69	32.02	38.22	<0.01
Acute Respiratory Failure	36.06	33.91	37.3	0.20
**Resource Use**				
LOS, d	5.51	5.71	5.78	0.44
Hospital cost, $	84,336	57,516	85,509	0.38
**Advanced Procedures (%)**				
Implantable defibrillator	0.82	1.1	1.16	0.24

CRT	0.37	0.39	0.53	0.47
Ventricular assist device	0.17	0.63	0.37	0.03
Heart Transplant	0.37	0.16	0.35	0.52

Table S3. Propensity matched analysis results for racial disparities for major outcomes in heart failure patients.

**Variable**	**Whites**	**Blacks**	**Hispanics**	***P* value***
Deaths	*Ref*	0.76	0.79	<0.01
Cardiogenic shock	*Ref*	0.88	0.73	<0.01
Cardiac arrest	*Ref*	1.22	1.39	<0.01
Acute Kidney Injury	*Ref*	1.18	1.09	<0.01
Acute Respiratory Failure	*Ref*	0.77	0.79	<0.01

Table S4. Cox proportional Hazards regression results for hospital mortality by racial groups.

**Mortality**	**Adjusted HR**	**Confidence Interval**	***P* value**
Blacks vs. Whites (ref)	0.76	0.70-0.83	<0.01
Hispanics vs. Whites (ref)	0.82	0.73-0.93	<0.01

HR represents Hazard ratio.
